# X-ray Crystallographic Study of Preferred Spacing by the NF-κB p50 Homodimer on κB DNA

**DOI:** 10.3390/biom13091310

**Published:** 2023-08-26

**Authors:** Norman Zhu, Matthew Mealka, Shane Mitchel, Christy Milani, Lisa M. Acuña, Eric Rogers, Ashlee N. Lahana, Tom Huxford

**Affiliations:** Structural Biochemistry Laboratory, Department of Chemistry & Biochemistry, San Diego State University, 5500 Campanile Dr., San Diego, CA 92182-1030, USA; zhunorman@gmail.com (N.Z.); mealka.matthew@gmail.com (M.M.); shanemitchel97@gmail.com (S.M.); casd199@gmail.com (C.M.); lisa.acuna23@gmail.com (L.M.A.); rogers85748@gmail.com (E.R.); ashlee.lahana@quidelortho.com (A.N.L.)

**Keywords:** DNA, IκBζ, NF-κB, protein–DNA complex, p50, transcription factor, X-ray crystallography

## Abstract

Though originally characterized as an inactive or transcriptionally repressive factor, the NF-κB p50 homodimer has become appreciated as a physiologically relevant driver of specific target gene expression. By virtue of its low affinity for cytoplasmic IκB protein inhibitors, p50 accumulates in the nucleus of resting cells, where it is a binding target for the transcriptional co-activator IκBζ. In this study, we employed X-ray crystallography to analyze the structure of the p50 homodimer on κB DNA from the promoters of human interleukin-6 (IL-6) and neutrophil-gelatinase-associated lipocalin (NGAL) genes, both of which respond to IκBζ. The NF-κB p50 homodimer binds 11-bp on IL-6 κB DNA, while, on NGAL κB DNA, the spacing is 12-bp. This begs the question: what DNA binding mode is preferred by NF-κB p50 homodimer? To address this, we engineered a “Test” κB-like DNA containing the core sequence 5′-GGGGAATTCCCC-3′ and determined its X-ray crystal structure in complex with p50. This revealed that, when presented with multiple options, NF-κB p50 homodimer prefers to bind 11-bp, which necessarily imposes asymmetry on the complex despite the symmetry inherent in both the protein and its target DNA, and that the p50 dimerization domain can contact DNA via distinct modes.

## 1. Introduction

NF-κB is a family of evolutionarily conserved transcription factors that provide stimulus-dependent temporal control over target gene expression [1,2,3]. In mammals, functional NF-κB dimers regulate transcription for developmental and adaptive immune programs as well as orchestrate immediate and prolonged gene expression responses to diverse pro-inflammatory stimuli [4,5,6]. This latter feature has resulted in NF-κB becoming an important system for understanding inflammatory disease progression and development of chronic syndromes, such as diabetes and cancer [7].

NF-κB p50 was originally identified as one of two protein subunits, together with RelA (p65), that comprise the inducible heterodimeric transcription factor NF-κB [8]. Identification of the gene responsible for the functioning p50 subunit revealed that it is, in fact, the product of incomplete proteolysis of the p105 protein product of the *NFKB1* gene [9]. Processing of p105 to p50 occurs constitutively in cells, and induction of the IκB kinase (IKK) activity signals complete degradation of p105 with further release of associated p50 protein [10,11]. This results in the availability of mature p50, which bears the signature features of subunit dimerization, nuclear localization, and sequence-specific DNA binding. In contrast to other mammalian NF-κB subunits, such as RelA, RelB, and c-Rel, the p50 subunit (as well as p52) lacks transcriptional activation potential, which, in the other NF-κB subunits, depends upon the presence of their carboxy-terminal transcription activation domains.

Despite its original characterization as a repressor of target gene transcription, the NF-κB p50 homodimer was subsequently shown to be an active transcription factor through its association with other nuclear factors, such as the IκBζ co-activator protein [12]. In resting cells, NF-κB dimers with inherent potential to activate target gene expression are retained in the cytoplasm as inactive complexes with a member of the IκB family of inhibitor proteins. In classical NF-κB signaling, for example, the p50:RelA heterodimer associates noncovalently with IκBα. This interaction relies primarily on the RelA subunit, and, consequently, homodimers of p50 escape cytoplasmic retention by IκBα and localize to the nucleus in resting cells, where, by virtue of their potential for sequence-specific DNA binding, they associate at promoters or enhancers of target genes.

Induction of cytoplasmic NF-κB by stimuli such as interleukin-1 (IL-1) or bacterial lipopolysaccharide (LPS) leads to rapid phosphorylation- and ubiquitin-dependent proteolytic removal of IκBα and accumulation of p50:RelA heterodimer in the nucleus. The early products of classical NF-κB signaling typically include inflammatory cytokines and effectors as well as the IκBα inhibitor protein and, in some cell types, a structurally related protein known as IκBζ [13]. Both newly synthesized IκBα and IκBζ proteins find their way into the nucleus, where IκBα is capable of disrupting p50:RelA:DNA complexes and directing subcellular localization to the cytoplasm, thus restoring the inactive state. Nuclear IκBζ, on the other hand, interacts specifically with NF-κB p50 subunits, forming a ternary complex on DNA [14]. In mice, ablation of the gene encoding IκBζ blocked delayed induction of interleukin-6 (IL-6) in peritoneal macrophages in response to toll-like receptor 4 (TLR4) stimulation [15]. IκBζ has also been implicated in directing p50-dependent expression of the human neutrophil-gelatinase-associated lipocalin (NGAL) gene [16]. Although the precise mechanism(s) through which IκBζ works with p50 to elevate expression of select NF-κB target genes remain(s) unclear, studies in murine macrophages showed that IκBζ forms a complex with Akirin2 to recruit the SWI/SNF chromatin remodeling complex to the IL-6 promoter in response to TLR stimulus [17].

In order to determine whether binding to IκBζ-responsive κB DNA generates any unique structural signatures that might be specifically recognizable to nuclear IκBζ, we crystallized and determined the X-ray co-crystal structures of the DNA-binding Rel homology region (RHR) of murine NF-κB p50 homodimer in complex with κB DNA taken from the promoters of IκBζ-responsive genes interleukin-6 (IL-6) and neutrophil-gelatinase-associated lipocalin (NGAL). We also report the X-ray co-crystal structure of the p50 homodimer RHR in complex with an engineered “Test” DNA containing the symmetrical κB-like DNA sequence 5′-GGGGAATTCCCC-3′. Taken together, and in comparison with previously determined X-ray crystal structures of p50 homodimer in complex with κB DNA, the crystallographic models suggest a preferred mode of DNA binding by the NF-κB p50 homodimer that necessarily introduces asymmetry into the resulting complex and allows for movement of the p50 dimerization domains between alternative positions relative to the bound DNA.

## 2. Materials and Methods

### 2.1. DNA Oligonucleotides

Single-stranded DNA oligonucleotides were purchased from IDT (San Diego, CA, USA). Freeze-dried oligos (~1 μg) were resuspended in 0.8 mL low salt buffer (0.01 M NaOH, 0.1 M NaCl), centrifuged (Thermo Fisher Scientific, Waltham, MA, USA) at 16,000 rpm for 5 min, loaded on to a Source 15Q 10/10 anion exchange column in 90% low salt buffer and 10% high salt buffer (0.01 M NaOH, 1 M NaCl). The column was washed with two column volumes and eluted via a linear gradient from 10% to 90% high salt buffer over ten column volumes. Peak fractions were combined and neutralized via addition of 12.5 μL 0.5 M Na-MES pH 6.5 per mL DNA. Concentrations were determined via absorbance at 260 nm wavelength, and complementary oligos were mixed at a 1:1 molar ratio, placed on a 96 °C heat block, and allowed to anneal via slow cooling for 20 min. Double-stranded oligos were then diluted with four volumes of ultra-pure water and applied to a 2 mL Q Sepharose Fast Flow ion exchange column (Cytiva Life Sciences, Marlborough, MA, USA), washed with 10 column volumes of water, and eluted in 4 mL 1 M NaCl solution. Finally, the DNA was concentrated in a centricon-3 centrifugal concentration unit with dilution in water until a final concentration of ~2 mM and stored in frozen aliquots at −20 °C.

### 2.2. Protein Expression and Purification

Recombinant expression and purification of the murine NF-κB p50 subunit Rel homology region consisting of amino acid residues 39–363 has been reported previously [14]. Briefly, a pET11a plasmid bearing the cDNA under an inducible phage T7 promoter was introduced into chemically competent *E. coli* bacterial strain BL21 (DE3) via heat shock transformation and cultured at 37 °C in 2 L LB media supplemented with 100 μg/mL ampicillin. Bacteria cells were isolated via centrifugation, resuspended in lysis buffer (25 mM Na-MES pH 6.5, 50 mM NaCl, 10 mM β-mercaptoethanol, 0.5 mM ethylenediaminetetraacetic acid, and 0.5 mM phenylmethylsulfonyl fluoride), and homogenized via passage through an M110L model Microfluidizer (Microfluidics, Westwood, MA, USA). Lysates were clarified via centrifugation at 12,000 rpm in an SS-34 rotor. Nucleic acid was precipitated from clarified lysates with slow addition of 10% (*w*/*v*) streptomycin sulfate to a final concentration of ~0.5% and stirring for 20 min at 4° C followed by a second centrifugation. The supernatant containing the soluble protein fraction was next passed through 0.8 μm syringe-tip filter and applied via gravity through a 20 mL SP Sepharose Fast Flow column. The column was washed with 10 column volumes of lysis buffer and eluted via linear gradient of 50 to 500 mM NaCl over 20 column volumes. Eluted fractions were analyzed by SDS-PAGE and fractions containing p50 protein were combined and concentrated to ~5 mL total volume in centriprep-10 centrifugal concentrators (MilliporeSigma, Rockville, MD, USA). The protein was further purified by size exclusion chromatography through a Superdex 75 16/60 gel filtration column in 25 mM Tris-HCl pH 7.4, 50 mM NaCl, 1 mM DTT. Peak fractions were combined and concentrated via centrifugation in a centricon-10 to ~15 mg/mL, flash frozen in liquid nitrogen, and stored in 25 μL aliquots at −80 °C.

### 2.3. NF-κB p50:DNA Complex Formation and Co-Crystallization

For p50:IL-6 complexes, 17-mer DNA from the human IL-6 gene promoter was combined with p50 RHR protein at a molar excess of 1.1:1 DNA:protein. The mixture was allowed to equilibrate at room temperature for at least five minutes and complex co-crystals were prepared via the hanging drop vapor diffusion method in which 1 μL complex was mixed with 1 μL reservoir solution (0.1 M Na-Acetate pH 4.9, 16% (*w*/*v*) PEG 1500) on siliconized cover glass and sealed over 1 mL reservoir solution. Hexagonal crystals of roughly 0.1 mm in each dimension grew within roughly seven days at room temperature.

To prepare p50:(NGAL)_2_ complexes, 10-mer NGAL κB DNA was combined with p50 RHR at a 2:1 ratio of DNA:protein. Complex co-crystals were grown via hanging drop vapor diffusion against 0.05 M Na-Acetate pH 5.4, 20 mM MgSO_4_, and 4% PEG 3350. Rod-like crystals appeared overnight at room temperature and reached their full size after 48 h.

To form p50:Test complexes, 16-mer Test DNA with 5′-T single base overhangs was combined at a 1:1 ratio of DNA:protein with p50 RHR homodimer. Complex co-crystals were grown via sitting drop vapor diffusion with a TTP Labtech Mosquito robot. Further, 100 nL of complex was combined with 100 nL reservoir solution containing 4% Tacsimate pH 6.0, 12% PEG 3350, and incubated over a sealed well containing 59.9 μL reservoir solution. Single box-like crystals of roughly 0.1 mm on each edge grew after 1 week incubation at 20 °C.

### 2.4. X-ray Diffraction Data Collection

The p50:IL-6 complex crystals were harvested with nylon loops and transferred to a cryo protectant solution containing 0.1 M Na-Acetate pH 5.9, 20% PEG 1500, and 25% (*v*/*v*) glycerol. After that, they were flash-cooled via plunging into liquid nitrogen. Complete X-ray diffraction data were collected on an ADSC Q315 CCD detector at NSLS beamline X25 (Table 1). Data were processed in HKL2000 (HKL Research, Charlottesville, VA, USA) [18].

Crystals of p50:(NGAL)_2_ complex were transferred and flash-cooled in 50% Paratone N, 50% 2-methyl-2,4-pentanediol. Data collection was performed using an ADSC Q315R CCD detector at ALS beamline 8.2.1. Data were processed in HKL2000 [18].

The p50:Test complex co-crystals were stabilized and flash-cooled in 4% Tacsimate pH 6.0, 15% PEG 3350, and 25% ethylene glycol. Diffraction data were collected on a Dectris Pilatus 6 M detector at APS beamline 24-ID-C (Table 1). Data were processed in XDS (Manufacturer, City, State abb if USA or Canada, Country and software version on first mention) [19].

### 2.5. Structure Solution and Refinement

The p50:IL-6 complex crystal structure was solved by molecular replacement using one copy of the dimerization domain complex (amino acids 248–350) and two copies of the amino-terminal domain (amino acids 39–239) from the p50:DNA complex crystallographic model (PDB accession code: 1NFK) as probes [20]. Molecular replacement in PHASER against working data between 50–3.30 Å data provided a clear solution with *Z*-score of 19.0 and log-likelihood gain (LLG) of 1768 [21]. Similar approaches using domains from the refined p50:IL-6 complex structure as probes were employed for first estimates of phase via molecular replacement on p50:(NGAL)_2_ and p50:Test complex data. For all three complexes, model-building was performed in COOT and maximum likelihood refinement was carried out in PHENIX [22,23]. Model stereochemistry and validation were monitored in MolProbity [24].

**Table 1:** X-ray crystallography data collection and refinement parameters.

	p50:IL-6	p50:(NGAL)_2_	p50:Test
*Data Collection*			
X-ray source	NSLS X25	ALS 8.2.1	APS ID-24-C
Wavelength (Å)	1.0000	0.9999	0.9792
Space group	P6_5_	P2_1_2_1_2_1_	P2_1_
Unit cell (Å)			
a	162.24	68.07	47.15
b	162.24	103.34	146.45
c	60.43	128.50	67.69
α, β, γ (°)	90, 90, 120	90, 90, 90	90, 98.62, 90
Complex/asymm. unit	1	1	1
Resolution range (Å) ^1^	50–2.80 (2.85–2.80)	50–2.80 (2.85–2.80)	146.45–3.00 (3.16–3.00)
*R*_sym_ (%)	7.9 (59.4)	8.3 (76.4)	9.4 (63.1)
Observations	112,366	138,228	61,285
Unique reflections	22,645	23,123	17,962
Completeness (%)	100 (100)	100 (99.9)	98.6 (99.1)
Redundancy	5.0 (5.0)	6.0 (6.1)	3.4 (3.5)
<*I*/σ>	17.9 (2.0)	19.0 (2.3)	10.1 (2.0)
*Refinement*			
Number of reflections	22,567 (2238)	21,798 (1749)	17,018
*R*_work_ (%)	22.4 (38.8)	23.8 (30.0)	23.6 (33.9)
*R*_free_ (%) ^2^	27.2 (40.1)	28.7 (35.8)	30.8 (42.2)
Protein/DNA/H_2_O atom	4903/690/55	4906/805/0	4906/691/0
Geometry (R.m.s.d.)			
Bond lengths (Å)	0.003	0.004	0.003
Bond angles (°)	0.630	0.594	0.587
Mean *B* (Å^2^)	60.3	81.3	84.1
Ramachandran plot ^3^			
Favored (%)	91.5	93.6	93.4
Allowed (%)	7.8	5.7	5.9
Outliers (%)	0.7	0.7	0.7
MolProbity score ^4^	1.84	1.78	1.78
PDB accession code	8TKM	8TKN	8TKL

^1^ Data in parentheses are for highest-resolution shell. ^2^ Calculated against a cross-validation set of at least 5% of data selected at random prior to refinement. ^3^ Calculated by MolProbity [24]. ^4^ Combines clashscore, rotamer, and Ramachandran evaluations to a single score, normalized to the same scale as X-ray resolution [24].

### 2.6. Structure Analysis and Figures

Structural figures as well as distance measurements, superpositions, and r.m.s.d. calculations were all executed in PyMol (Schrödinger, New York, NY, USA) [25]. DNA conformational analysis was performed using Web 3DNA 2.0 [26].

## 3. Results

### 3.1. NF-κB p50 Homodimer:IL-6 κB DNA Complex Co-Crystal Structure

**Figure 1:**
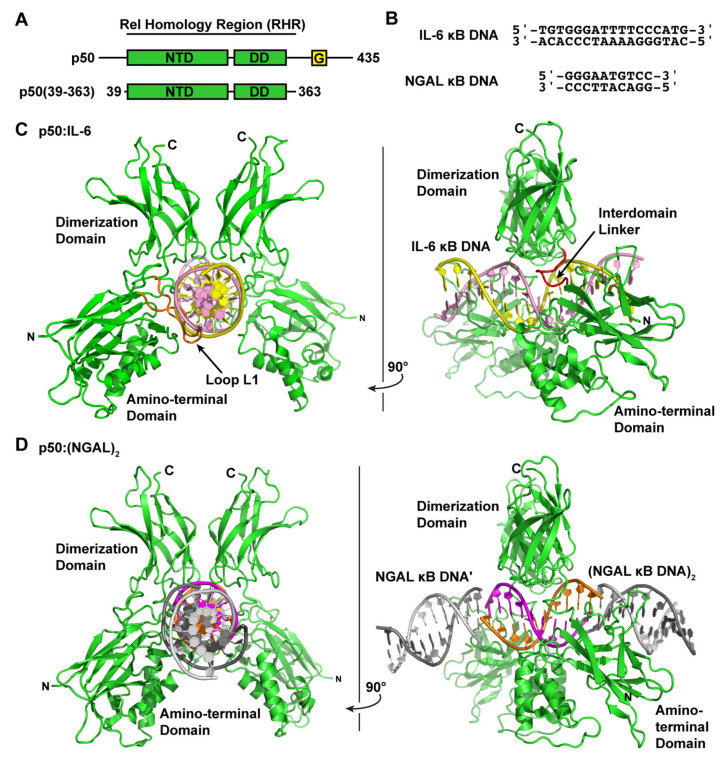
X-ray crystal structures of the NF-κB p50 homodimer Rel homology region (RHR) bound to κB DNA. (**A**) A domain map of the mature mammalian p50 subunit with the amino-terminal domain (NTD), dimerization domain (DD), and glycine-rich region (G) labeled. The p50 RHR protein construct employed in the crystallographic studies is depicted schematically below. (**B**) Sequences of the double-stranded IL-6 and NGAL κB DNA employed in the crystallographic studies. (**C**) Ribbon diagram representation of the X-ray crystal structure of p50 RHR homodimer bound to the IL-6 κB DNA viewed down the long DNA axis (left) and after 90° rotation about the *y*-axis (right). The individual p50 RHR subunits are colored green and the two DNA strands are pink and yellow. (**D**) The X-ray crystal structure of the p50 RHR (green) bound to NGAL κB DNA (magenta and orange) viewed as in (**C**). The p50 homodimer contacts one base at each end of a second NGAL κB DNA (colored grey) contained within the crystallographic asymmetric unit. Two crystallographically equivalent copies of this second NGAL κB DNA are included in the figure.

The pluripotent inflammatory cytokine IL-6 has long been known to be an NF-κB-dependent gene [27,28]. IL-6 has been shown, both in mouse knockout and cell-based studies, to be responsive to classical NF-κB-inducing stimuli, such as IL-1 or LPS, in an IκBζ-dependent manner [15]. Therefore, in order to directly observe the NF-κB p50 homodimer in complex with κB DNA from an IκBζ-dependent gene promoter, we crystallized the murine-DNA-binding Rel homology region (RHR) from NF-κB p50 (amino acids 39–363) in complex with a blunt-ended 17-mer double-stranded DNA sequence from the promoter of the human interleukin-6 (IL-6) gene (Figure 1A,B). The DNA contains the consensus κB DNA sequence 5′-GGGATTTTCCC-3′ that, according to our present understanding of DNA recognition by NF-κB, is targeted preferentially by p50 homodimer [29,30]. Previous in vitro studies of murine p50 homodimer RHR binding to human IL-6 κB DNA via electrophoretic mobility shift assay (EMSA) revealed an equilibrium dissociation constant (*K*_D_) of ~10 nM [14].

We determined the X-ray co-crystal structure of an NF-κB p50 homodimer:IL-6 κB DNA complex (p50:IL-6) at 2.80 Å resolution (Table 1). The resulting crystallographic model reveals the well-characterized DNA-binding NF-κB RHR (also commonly referred to as the “RHD”) composed of an amino-terminal domain (murine p50 residues 39–239) and a short interdomain linker (residues 240–247) followed by the dimerization domain (residues 248–350) (Figure 1C). As is the case in several other NF-κB:DNA complex crystal structures, p50 amino acid residues 351–363, which contain the nuclear localization signal, are disordered within the crystal and, therefore, do not contribute to diffraction and are not contained in the crystallographic model.

Consistent with previously studied p50:DNA complexes, base-specific contacts with IL-6 κB DNA are mediated primarily by amino acids within loop L1 (amino acids 47–79) of the p50 subunit amino-terminal domain. His64, Arg56, and Arg54 predictably mediate hydrogen bond contacts with each of three guanine bases at the 5′-ends of the embedded κB DNA, and Glu60 interacts with cytosine from the third G:C base pair (bp) of these signature triplet G bases [31]. Additional base-contacting hydrogen bond interactions are mediated by Lys241 from the interdomain linker. As a consequence of this preference by p50 to bind to three consecutive G bases, the p50 homodimer binds to IL-6 κB DNA with 11-bp spacing as two separate 5-bp half sites separated by a central T:A bp.

With respect to its potential for binding to IκBζ, our p50:IL-6 crystallographic model alone does not offer a clear indication of how a ternary IκBζ:p50:DNA complex might assemble. As has been illustrated previously via structural studies based on X-ray crystallographic analyses, interaction of DNA-bound NF-κB with classical IκBα or IκBβ or binding of the nuclear IκB protein Bcl-3 through a similar mode would necessarily result in collision with the DNA absent of any structural rearrangement of protein and/or nucleic acid [32,33,34,35].

### 3.2. NF-κB p50 Homodimer:NGAL κB DNA Complex Co-Crystal Structure

The human neutrophil-gelatinase-associated lipocalin (NGAL) gene was reported to show particularly robust expression upon co-transfection of NF-κB p50 and IκBζ in cultured HEK 293 cells [16]. Subsequent studies have revealed that epithelial cell NGAL expression in response to inflammation is controlled exclusively by p50 and IκBζ [36]. Original characterization of NGAL as an NF-κB-dependent gene in A549 lung carcinoma epithelial cells identified the unique sequence 5′-GGGAATGTCC-3′ from within the NGAL gene promoter as a bona fide κB DNA site [37]. This 10-bp sequence contains three consecutive G bases at the 5′-end but only two C bases at the 3′, which is typical of κB DNA that preferentially binds to NF-κB p50:RelA heterodimers [31]. We do note, however, that a third C base occupies the eleventh position within the human NGAL promoter, which is consistent with preferential binding to p50 homodimer [30]. In order to observe whether binding to the NGAL promoter caused noticeable structural changes that might promote association with IκBζ, we crystallized and determined the X-ray crystal structure of the complex of NF-κB p50 homodimer and the 10-bp NGAL κB DNA to 2.80 Å resolution. Crystals were only obtained when complexes were prepared with a 2:1 molar ratio of NGAL κB DNA:p50 homodimer. Therefore, it was not completely surprising to observe that the 10-mer NGAL κB DNA molecules orient themselves end-to-end in a continuous manner throughout the crystal with one NGAL κB DNA bound up entirely by p50 homodimer and another identical NGAL κB DNA almost completely unbound in the crystallographic asymmetric unit (Figure 1D). To indicate that this is the case, we refer to the resulting complex as “p50:(NGAL)_2_.”

Interestingly, although the base-specific contacts of p50 to DNA in the p50:(NGAL)_2_ complex crystallographic model are practically identical to the interactions between p50 and DNA in the p50:IL-6 crystal structure, the spacing between p50 subunits on the DNA differs. On the NGAL κB DNA, p50 interacts with one G base each from the otherwise unbound NGAL κB DNA “neighbors.” One of these double-stranded DNA molecules is part of the crystallographic asymmetric unit, while the other is crystallographically identical, though part of a different asymmetric unit (Figure S1). The result is that, on the 10-mer NGAL κB DNA, p50 homodimer binds effectively with 12-bp spacing (two 5-bp half sites with a central AT:TA duplex of base pairs).

Due to the fact that they are not joined by phosphodiester linkages, the NGAL κB DNA molecules approximate but do not exactly assemble into a continuous B-DNA double helix. Consequently, the first and last G bases of the unbound DNA molecules occupy positions directly over the G bases at the ±3 positions in the bound NGAL κB DNA, as if they were rotated back two positions in a left-handed helical fashion (Figure S1). Nonetheless, His64 from loop L1 of p50 remains capable of contacting these misplaced G bases without disrupting the ability of Arg 56, Arg54, and Glu60 to also contact their bases. This illustrates the versatility of the loop L1 base-contacting amino acids in binding to DNA with altered conformations. A similar example of loop L1 amino acids accommodating significant changes while maintaining the ability to interact with unique nucleic acid base sequences was observed previously in the X-ray crystal structure of NF-κB p50 homodimer in complex with an in vitro- and in vivo-selected high-affinity RNA aptamer [38,39].

### 3.3. Comparative Analysis of DNA Base Contacts in p50:DNA Complex Co-Crystal Structures

**Figure 2:**
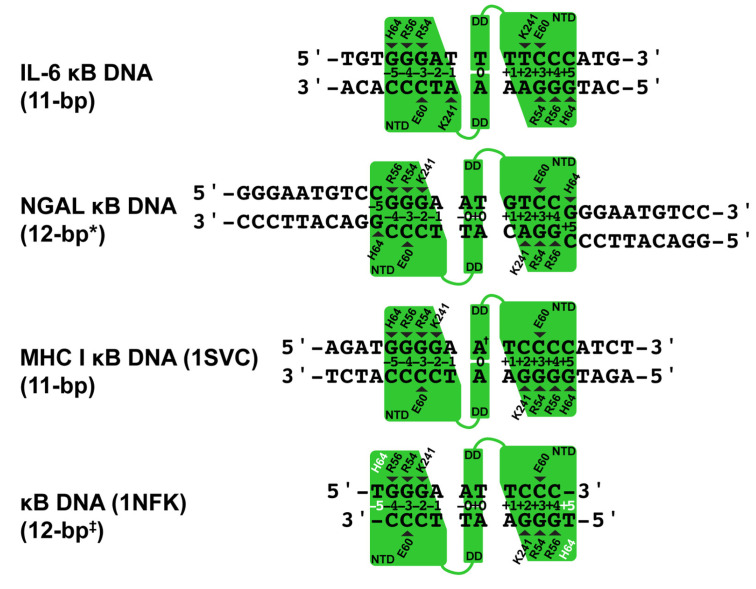
Schematic representation of base-specific contacts to κB DNA in p50:DNA complex crystal structures. The p50 homodimer binds to the IL-6 κB DNA with 11-bp spacing. Base-specific contacts from loop L1 residues His64, Arg56, Arg54, and Glu60 as well as Lys241 from the interdomain linker are illustrated by triangles that point to the base contacted. NGAL κB DNA is bound with 12-bp spacing, where * denotes that two of the bases are contributed by terminal 5′-G bases from two otherwise unbound crystallographically equivalent DNA neighbors. MHC I κB DNA is bound with 11-bp spacing, where ^†^ marks the central A:A mismatch that is contained within this complex crystal structure. The 1NFK complex crystal structure binds to a 10-mer κB DNA with effective 12-bp spacing ^‡^ on account of two His64 amino acids (white text) that fail to make contact with DNA due to the length of κB DNA employed in that study.

Our two X-ray crystal structures reveal that the NF-κB p50 homodimer binds with 11-bp spacing within the 17-mer DNA sequence taken from the human IL-6 promoter containing its κB DNA. As a consequence of its interaction with G bases at the ends of neighboring unbound NGAL κB DNA, the same p50 homodimer binds with an effective spacing of 12 bp to the 10-mer κB DNA sequence from the promoter of the human NGAL gene (Figure 2). This difference closely resembles the original observations made in 1995 when the first X-ray crystal structures of the NF-κB p50 RHR in complex with κB DNA were reported [20,40]. One model (PDB ID: 1SVC) contained the human p50 homodimer RHR bound with 11-bp spacing to κB DNA from the MHC Class I gene complex. It bears mentioning that human and mouse p50 proteins are practically identical, differing at only seven non-critical amino acid positions throughout the entire RHR. For the sake of consistency with most published work, in this study, we shall continue to use the murine amino acid numbering. It should also be noted that the DNA in that study contained an A:A mismatch at its central position, giving the 1SVC complex structure the unique property of being truly chemically equivalent about a two-fold rotation axis of symmetry. The other p50:DNA complex X-ray crystal structure (1NFK) reported binding with 10-bp spacing to a symmetrical κB DNA with the sequence 5′-GGGAATTCCC-3′. Analysis of base-specific contacts reveals that the 1NFK model, in fact, exhibits the same 12-bp spacing as p50:(NGAL)_2_, the main difference being that, in 1NFK, the shorter 10-mer κB DNA target contains an insufficient number of base pairs to support contact with His64 residues from each of the two p50 subunits.

A detailed analysis of the base-specific contacts between NF-κB p50 homodimer and κB DNA reveals the importance of loop L1 residues in DNA sequence selectivity (Figure 2). As has been previously reported, p50 residues His64, Arg56, and Arg54 preferentially contact three neighboring G bases with Glu60, forming a hydrogen bond with the C base paired with the third G [31]. Exceptions to this rule are observed in the p50:(NGAL)_2_ and 1NFK models, wherein both bound κB DNA molecules contain only 10 bp each. In the case of p50:(NGAL)_2_, this is resolved by two unbound blunt-end DNAs that pack at the ends of the bound κB DNA target. Both of these neighbors contain G bases at their ends that satisfy the preference for His64 binding, even though, as mentioned previously, those G bases are in different positions than if they were actually part of the NGAL κB DNA target. The result is that p50 binds NGAL κB DNA with 12-bp spacing. The κB DNA in 1NFK contains 5′-T single-base overhangs, which impede DNA end-to-end packing. Consequently, p50 must either compress to bind with 10-bp spacing or give up binding through His64. It is interesting to observe that the solution is for both p50 subunits to surrender base contact via His64, resulting in a symmetrical complex with effective 12-bp spacing.

The p50 homodimer binds κB DNA very similarly with 11-bp spacing in both p50:IL-6 and 1SVC complexes (Figure 2). The key difference is that, due to its palindromic sequence and central A:A base mismatch within the 19-mer DNA containing the MHC I κB DNA sequence, 1SVC exhibits perfect internal two-fold rotation symmetry while the two halves of the p50:IL-6 complex, although closely similar, are nonetheless chemically distinct.

Another notable difference in DNA base sequence recognition by p50 involves Lys241 from the interdomain linker of p50. Although it appears to clearly prefer binding to a fourth G base, when that is not an option, Lys241 can move to accommodate either base of an A:T bp on either DNA strand, and even, as illustrated by subunit A of the p50:IL-6 complex, from a different position within the κB DNA.

### 3.4. X-ray Co-Crystal Structure of NF-κB p50 Homodimer in Complex with “Test” κB DNA

**Figure 3:**
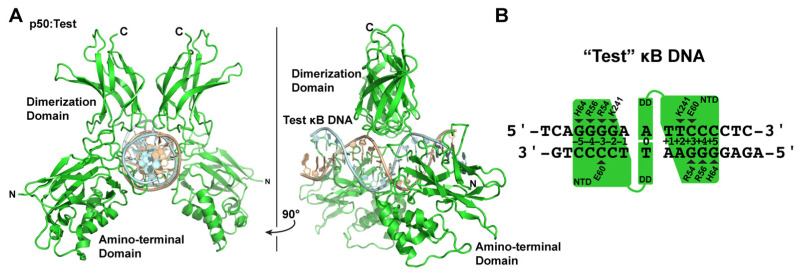
X-ray crystal structure of the NF-κB p50 homodimer RHR bound to a “Test” κB-like DNA. (**A**) Ribbon diagram representation of p50 RHR homodimer (green) bound to 16-mer DNA (light cyan and wheat) viewed down the long DNA axis (left) and rotated 90° about the *y*-axis (right). (**B**) Base-specific contacts to κB DNA in the p50:Test complex crystal structure, depicted schematically as in Figure 2, reveal that p50 binds with 11-bp spacing.

The various X-ray crystallographic structures of NF-κB p50 homodimer in complex with κB DNA raise questions with respect to what actually is the preferred mode of κB DNA binding by p50, and does that offer any insight into how such complexes might be selectively targeted by IκBζ? To address these questions, we engineered a “Test” DNA containing the core κB-like sequence 5′-GGGGAATTCCCC-3′. We embedded this core sequence within ends of different sequence and length so as to impose asymmetry in crystal packing and unambiguous identification of DNA orientation during model-building and crystallographic refinement and determined the X-ray crystal structure of the resulting 16-mer DNA in complex with p50 at 3.0 Å resolution (Figure 3A).

The resulting crystallographic model clearly reveals that p50 binds the Test κB-like DNA with 11-bp spacing. Moreover, since the Test DNA does not contain a central A:A mismatch, the binding is inherently asymmetric. Base-specific contacts between p50:Test and p50:IL-6 are largely conserved (Figure 3B). One notable difference is that, in the p50:Test complex, Lys241 from subunit A binds to the available fourth G base, as expected. Lys241 from subunit B contacts the T base of a T:A bp, just as it does in the p50:IL-6 complex. This is significant because, if the p50 homodimer bound DNA with 12-bp spacing, then it could accommodate both triplet G binding by the loop L1 residues and a second favored Lys241:G interaction by subunit B. Rather, p50 binds to the Test κB DNA with 11-bp spacing. Therefore, the p50:Test complex X-ray crystal structure strongly suggests that 11-bp spacing to κB DNA is the preferred binding mode by the NF-κB p50 homodimer. Furthermore, it illustrates how the preferred binding of a two-fold symmetric p50 homodimer dimer to 11-bp within a two-fold symmetric κB DNA necessarily introduces asymmetry into an otherwise symmetrical complex. This structurally and chemically distinguishes one DNA half-site from the other and could provide directionality to the complex in a way that influences its association with co-activator proteins such as IκBζ.

### 3.5. DNA Is Not Altered Significantly by 11- or 12-Base Pair Spacing

**Figure 4:**
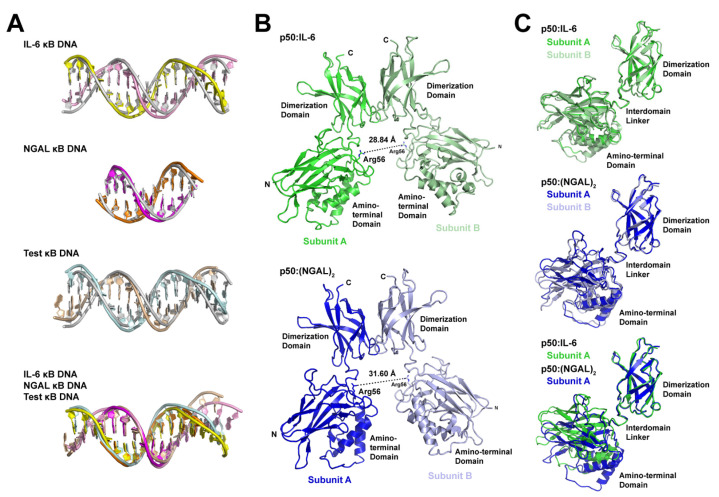
Structural analysis of the basis for alternative spacing by the NF-κB p50 homodimer RHR on κB DNA. (**A**) Superposition of κB DNA from p50:IL-6 (top), p50:(NGAL)_2_ (second from top), and p50:Test (second from bottom) complex X-ray crystal structures onto standard form B-DNA bearing the same sequence. Also, superposition of the three κB DNA onto one another (bottom). Coloring of DNA models is consistent with Figure 1 and Figure 3, and B-DNA is grey. (**B**) Ribbon diagram representations of the p50 homodimer RHR only from the p50:IL-6 (above; green) and p50:(NGAL)_2_ (below; blue) complex crystal structures with the distance between DNA base-contacting Arg56 CZ atoms measured and shown as a dashed line. (**C**) Superposition of the dimerization domains from both p50 RHR subunits from p50:IL-6 (above; green and pale green) and p50:(NGAL)_2_ (middle; blue and light blue) reveal little by way of relative movement of their respective amino-terminal domains. Superposition of subunits A from p50:IL-6 (green) and p50:(NGAL)_2_ (blue) (below) demonstrate a roughly 15° rotation of the amino-terminal domains relative to the dimerization domains to accommodate 11- or 12-bp spacing on κB DNA.

In order to identify the structural basis for 11- versus 12-bp spacing by the NF-κB p50 homodimer on different κB DNA, we first performed a detailed structural analysis of κB DNA from the p50:IL-6, p50:(NGAL)_2_, and p50:Test complex X-ray crystal structures. The analysis suggests that p50 binding introduces relatively minimal alteration from ideal B-form DNA structure. DNA curvature upon p50 binding is minimal and localized to the central bp, and DNA base geometries are normal, with only modest deviations observed among the bases contacted by p50 subunit loop L1 residues. These same regions also exhibit slight amounts of major groove widening and narrowing of the minor groove (Table S1). The minimal degree of change in DNA structure upon p50 binding is clearly illustrated when DNA from each of the three p50:DNA X-ray crystal structures is superimposed via least-squares fit to ideal B-DNA of the same length and sequence (Figure 4A). 

This conclusion agrees with a previously published study of the 1SVC and 1NFK complexes that reported minimal change in DNA structure other than an increase from 10- to nearly 11-bp per helical turn of κB DNA upon binding by p50 homodimer due to a slightly expanded major groove and narrower minor groove [41]. We conclude that major structural change in κB DNA upon complex formation does not determine 11- or 12-bp spacing by p50.

### 3.6. Individual p50 Subunits Adopt Similar Conformations Regardless of Spacing on Target DNA

If DNA is not significantly altered to accommodate 11- or 12-bp spacing by p50, then protein conformational change must account for the differences. This point is clearly illustrated by measuring the distance between Arg56 residues from the two DNA-bound p50 subunits (Figure 4B). Arg56 is a universally conserved amino acid within loop L1 of all NF-κB family proteins that plays the vital role of contacting the ±4 G base within the κB DNA target. In the p50:IL-6 complex, which binds with 11-bp spacing, the distance between CZ atoms in Arg56 from the two p50 subunits is 28.84 Å. In the 12-bp-spaced p50:(NGAL)_2_ complex, that distance is 31.60 Å, or roughly an increase in the distance between neighboring base pairs in standard B-form DNA (Table 2). Similar values are observed for the 1SVC and 1NFK complex X-ray crystal structures with 11- and 12-bp spacing, respectively, and the distance between Arg56 side chains in our crystallographic model of p50 homodimer bound to the Test DNA with 11-bp spacing is 28.57 Å. This clearly indicates that change in the conformation of p50 homodimer is responsible for the differences in spacing observed upon p50 binding to κB DNA.

Analysis of the two p50 subunit dimerization domains from any of the three X-ray crystal structures quickly leads one to the conclusion that they are structurally indistinct and are, in fact, identical in structure to the isolated p50 dimerization domain complex independent of amino-terminal domains or bound DNA [42]. The individual p50 amino-terminal domains, despite their varied spacing, also exhibit only very slight structural differences when compared with one another. Therefore, the difference in conformation of individual p50 subunits with 11- or 12-bp spacing on κB DNA is a result of movement of the amino-terminal domain(s) relative to the dimerization domain by virtue of flexibility in the interdomain linker. The interdomain linker has been implicated in previous studies in allowing the p50 amino-terminal domain to move relative to its dimerization domain in order to accommodate nucleic acid binding [38,39,41,43]. Additional examples of this property are illustrated by the NF-κB RelA subunit to accommodate high-affinity binding to a κB DNA with only one half-site for base-specific DNA contact as well as in its complex with the IκBα inhibitor protein [32,33,44].

We next wished to analyze whether binding to κB DNA with 11- or 12-bp spacing necessarily induces structural asymmetry to the various p50 homodimers. To test this, we performed a least-squares fit and calculated the root-mean-square deviation (r.m.s.d.) for position of Cα atoms for each of the DNA complex-bound p50 subunits relative to others (Table 3). The results of this analysis showed that individual p50 subunits from complexes that have 11-bp spacing (p50:IL-6 and p50:Test) differ significantly from the p50 subunits from the 12-bp p50:(NGAL)_2_ complex. While this is not surprising, we do find it interesting that each p50 subunit superposes extremely well with its binding partner (subunit A versus subunit B). This indicates that, irrespective of whether the p50 homodimer binds with 11- or 12-bp spacing, both subunits of the complex settle into the same, or very closely similar, conformations regardless of the bases they contact in their respective DNA half site. The fact that the p50 subunit dimerization domain is structurally unchanged upon DNA binding allows it to be used as a reference from which to visually assess the degree with which amino-terminal domains move in the respective subunits upon binding κB DNA with 11- or 12-bp spacing. When the dimerization domain from subunit A from p50:IL-6 complex is used to superimpose the corresponding dimerization domain from subunit B, only very minimal change in the position of the two amino terminal domains is observed. The same is generally true for p50:(NGAL)_2_ complex subunits. However, comparison of p50 subunits from between both complexes illustrates drastic movement, including a roughly 15° rotation in the amino-terminal domain of the 11- versus 12-bp bound complexes (Figure 4C).

### 3.7. The p50 Homodimer Dimerization Domain Is a Versatile DNA Contacting Unit

**Figure 5:**
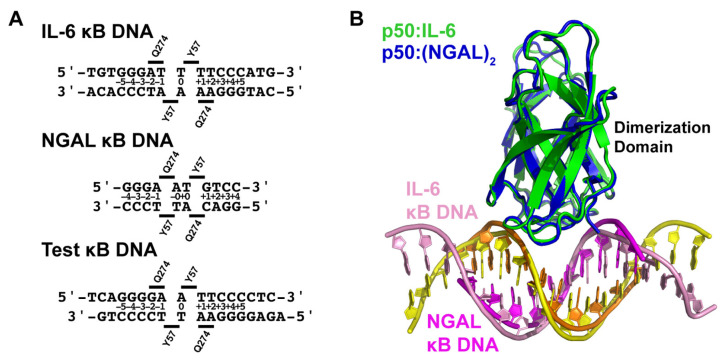
Alternative non-base-specific contacts with the DNA backbone allow the p50 homodimer dimerization domain to bind κB DNA via multiple modes. (**A**) Gln274 from the p50 dimerization domains accommodates differing positions to allow for both p50 subunits to adopt nearly identical conformations whether bound to κB DNA with 11-bp spacing (IL-6 and Test) or 12-bp spacing (NGAL). The position of Tyr57 from loop L1 of the p50 amino-terminal domain, a conserved residue that mediates important non-sequence-specific DNA backbone and base contacts, is included as a reference. (**B**) Ribbon diagram of the p50 subunit dimerization domain p50:IL-6 (green) and p50:(NGAL)_2_ (blue) and bound κB DNA (colored as in Figure 1). Superposition of the dimerization domains reveals that they contact their κB DNA differently (shifted by one-half of one bp) in the two complexes.

The observation that individual p50 subunits within a homodimer complex bind to κB DNA in more or less identical conformations regardless of whether the spacing between bound DNA bases is 11- or 12-bp necessarily implies that the dimerization domains must contact 11- and 12-bp κB DNA distinctly. As mentioned previously, the p50 dimerization domain exhibits an extremely stable fold that does not become altered regardless of its dimerization partner or upon binding to DNA or IκB. Importantly, although it makes considerable contact to DNA, the p50 dimerization domain does not directly contact DNA bases but rather contributes to p50:DNA binding stability through interactions with the DNA ribose-phosphate backbone. We analyzed DNA backbone contacts in the p50:IL-6, p50:(NGAL)_2_, and p50:Test complex X-ray crystal structures and observed marked differences in the relative positions of DNA contacting residues. For instance, Gln274 from the p50 dimerization domain contacts a backbone phosphate between nucleotides at the ±1 and ±2 positions in the p50:IL-6 and p50:Test complex structures with 11-bp spacing. However, in the p50:(NGAL)_2_ with 12-bp spacing, Gln274 binds between nucleotides ±0 and ±1 (Figure 5A). This difference places three bp between the two Gln274 residues in the eleven-bp-spaced p50:IL-6 and p50:Test complexes where only two bp separate Gln274 residues in the twelve-bp-spaced p50:(NGAL)_2_ complex (Figure 5A). This in turn changes the position of the central pseudo-dyad symmetry axis of the p50 dimerization domain by one half a base pair in the respective p50:DNA complexes (Figure S2A,B).

In order to visually illustrate this point, we superimposed the p50 dimerization domains (amino acids 248–350) from the p50:IL-6 and p50:(NGAL)_2_ complex crystal structures and observed that they overlap almost perfectly, even down to their DNA-backbone-contacting residues (Figure S2C). However, each of the base pairs in their respective bound κB DNA are shifted relative to one another by exactly one-half of a bp, illustratingthat the dimerization domains are capable of employing distinct modes of binding upon interaction with DNA (Figure 5B). This observation suggests that, by virtue of flexibility in the interdomain linker region, upon DNA binding, the p50 dimerization domains can move between alternative binding modes while the two amino-terminal domains remain anchored to their base-contacting binding sites. As binding with 11-bp spacing is likely to result in less tension and therefore greater flexibility in the interdomain linkers, we speculate that increased mobility of dimerization domains stabilizes p50 homodimer binding to DNA with 11-bp- relative to 12-bp spacing. This model for binding has implications for how the otherwise symmetrical NF-κB p50 homodimer might serve as a starting point for asymmetric multiprotein complex formation, even at symmetric κB DNA sites within the promoters of NF-κB target genes.

## 4. Discussion

The NF-κB p50 subunit shows a preference, both in cells and in vitro, for heterodimer formation with RelA subunits. However, either in the absence of RelA or when present in excess amounts, p50 readily assembles into homodimers [45]. These NF-κB p50 homodimers exhibit relatively low binding affinity for classical cytoplasmic IκB inhibitor proteins, such as IκBα or IκBβ [14,46]. As a consequence, p50 homodimers are observed in the nucleus of resting cells, where they bind to DNA [47]. More recently, unique roles for NF-κB p50 homodimer in directing specific target gene expression have been uncovered [48,49]. As mentioned previously, the nuclear IκB protein known as IκBζ has been shown to bind specifically to p50 homodimer, is capable of forming ternary complexes with p50 on DNA, and is required for the cell-specific expression of several NF-κB-dependent genes, including the pluripotent pro-inflammatory cytokine IL-6 and the siderophore-binding antimicrobial protein NGAL [15,16,36].

In light of this expanding role for NF-κB p50 homodimer in select target gene expression and in support of efforts to unravel a molecular mechanism for IκBζ-dependent NF-κB target gene expression, we endeavored to determine whether p50 binding to κB DNA from the promoters of IκBζ-dependent genes might provide insight into IκBζ:p50:DNA ternary complex formation. In this study, we determined X-ray co-crystal structures of the NF-κB p50 homodimer bound to a 17-mer DNA bearing the κB site from the promoter of human IL-6 and the 10-bp κB DNA from the human NGAL gene promoter. Our analysis of these crystallographic models in comparison with the two previously reported p50:DNA complex X-ray co-crystal structures reveals that p50 homodimer can bind to κB DNA with 11- or 12-bp spacing. In an attempt to identify which of the two binding modes is preferred, we crystallized and determined the X-ray co-crystal structure of NF-κB p50 homodimer in complex with an engineered “Test” κB-like DNA. As a 16-mer double-stranded DNA with 5′-T single nucleotide overhangs, the Test DNA is long enough to normalize any DNA end effects on binding. Furthermore, the two-fold symmetric κB-like DNA sequence is capable of supporting binding by p50 homodimer in a number of different symmetric or asymmetric arrangements and with varied spacing. This study identifies 11-bp as the preferred spacing by NF-κB p50 on DNA and suggests that introduction of asymmetry is a general feature of DNA binding by p50 homodimer.

Analysis of the crystallographic models determined for this study reveals that the ability of p50 to bind to κB DNA of similar sequence with 11- or 12-bp spacing is a result of differential placement of p50 subunit amino-terminal domains relative to the dimerization domains, afforded by flexibility inherent in the interdomain linker. Interestingly, regardless of κB DNA sequence, individual subunits of particular p50 homodimer:DNA complexes do not differ significantly from one another, as has been observed previously for NF-κB RelA homodimers in complex with κB DNA [44,50]. As we demonstrate in this study, the nearly identical conformations for the two p50 subunits in different complexes with DNA are maintained regardless of whether the homodimer exhibits 11- or 12-bp spacing. This appears to be a consequence of the ability of the structurally stable p50 dimerization domains to alternate between different modes of binding to the ribose-phosphate backbone of κB DNA. Maximization of alternative DNA binding modes by the p50 dimerization domains is a likely factor in stabilization of complexes with 11-bp spacing on DNA relative to 12-bp. Movement of the p50 dimerization domains relative to the DNA is a property that the dimerization domains are able to accomplish by virtue of the fact that, although they contain several conserved basic amino acids that contribute to overall complex stability, these residues do not contact specific bases of target DNA directly but rely entirely upon nonspecific electrostatic interactions with the DNA ribose-phosphate backbone.

**Figure 6:**
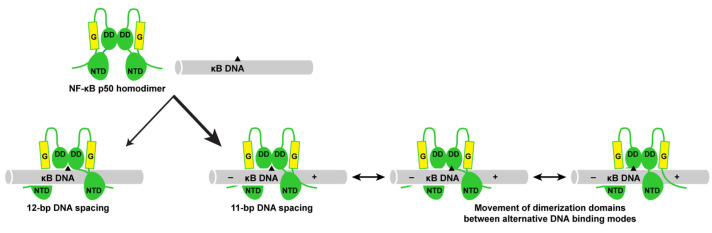
Schematic representation of structural modes for NF-κB p50 homodimer (RHR in green; glycine-rich region in yellow) binding to traditional κB DNA (grey with black triangle as a reference point). Complex formation can result in either 12- or 11-bp spacing of p50 on DNA. In both cases, the two p50 subunits are practically identical to one another, although the favored 11-bp spacing inherently introduces asymmetry into the complex (symbolized by “–” and “+” signs on κB DNA). The p50 subunit dimerization domains (“DD”) are capable of contacting DNA via alternative binding modes, allowing for their movement relative to the fixed amino-terminal domains (“NTD”). The combination of these factors might serve to support formation of asymmetric ternary IκBζ:p50:DNA complexes on the promoters of genes that rely on p50 and IκBζ for their elevated expression in response to stimuli.

The conclusions of this X-ray crystallographic study agree with previous in vitro experiments designed to identify consensus DNA sequences for NF-κB binding. Using the PCR-based SELEX method, Kunsch, et al. identified a consensus DNA sequence for p50 homodimer binding of 5′-GGGGATTCCC-3′ [29]. Nearly two decades later, Siggers, et al. employed an NF-κB-specific protein-binding DNA microarray to identify “traditional” 11- and 12-bp consensus DNA sequences for optimal p50 homodimer binding in addition to “non-traditional” 9-bp p50 homodimer binding sites [30]. It bears mentioning that the κB DNA promoters of IκBζ-responsive genes IL-6 and NGAL employed in this study are traditional κB DNA. In general, the traditional 11- and 12-bp p50 homodimer binding sites identified by Siggers, et al. are the same as the earlier consensus sequence of Kunsch, et al., with one 5′-G and/or 3′-C base added. Reevaluation of the Kunsch, et al. data, in fact, reveals that every 10-mer DNA generated in that earlier study via PCR from a pool of random 16 mers contained either a flanking 5′-G, a 3′-C, or both [29]. This provides strong evidence that p50 homodimers can and do bind κB DNA with both 11- and 12-bp spacing. Our experimental structural studies of p50 homodimer in complex with DNA of different sequences and lengths indicate that 11-bp spacing is preferred and that 11-bp spacing introduces unique features to p50:DNA complexes that could make them amenable for specific interaction with co-activator proteins (Figure 6).

We speculate that the preference for the NF-κB p50 homodimer to bind κB DNA asymmetrically with 11-bp spacing combined with the ability of the p50 dimerization domains to move between different DNA binding modes relative to the anchored amino-terminal domains provides the basis for a mechanism for selective binding of IκBζ to NF-κB p50 homodimers in the nucleus and generation of oriented, asymmetric ternary complexes to coordinate gene expression programs in response to specific NF-κB-inducing stimuli. However, even with asymmetric binding of p50 homodimer to establish directionality and movement of the dimerization domains to accommodate IκBζ, it remains likely that post-translational modification, additional binding co-factors, and/or DNA bending are required for stable ternary complex formation without collisions between IκBζ and DNA [51,52]. Further structural, biochemical, and cell-based transcriptional studies, including systematic analysis of the binding affinity of p50 homodimers for κB DNA that can accommodate 11- or 12-bp spacing, are required to more fully understand this system.

## Figures and Tables

**Table 2:** Distances between p50 subunits in complex with DNA.

p50:DNA Complex	Spacing on DNA	Distance (Å)
p50:IL-6	11-bp	28.84 ^1^
p50:(NGAL)_2_	12-bp	31.60
p50:Test	11-bp	28.57
1SVC	11-bp	28.71
1NFK	12-bp	32.36

^1^ Intersubunit distances between Arg56 CZ atoms in p50 homodimer:DNA complex X-ray crystallographic models.

**Table 3:** Structural similarity of NF-κB p50 subunits.

	IL-6 A	IL-6 B	NGAL A	NGAL B	Test A
**IL-6 A**	-	-	-	-	-
**IL-6 B**	1.056 ^1^	-	-	-	-
**NGAL A**	2.246	2.111	-	-	-
**NGAL B**	3.262	3.524	2.073	-	-
**Test A**	1.487	0.671	2.154	3.627	-
**Test B**	0.805	0.913	1.732	2.853	1.145

^1^ Root-mean-square deviation (r.m.s.d.) in units of Å for Cα positions after superposition of NF-κB p50 subunits (A or B) from p50:DNA complex crystal structures.

## Data Availability

Atomic coordinates and structure factor files for the p50:IL-6 DNA, p50:(NGAL)_2_ DNA, and p50:Test DNA complex crystal structures have been uploaded to the Protein Data Bank and assigned to accession numbers 8TKM, 8TKN, and 8TKL, respectively.

## Supplementary Materials

The following supporting information can be downloaded at: https://www.mdpi.com/article/10.3390/biom13091310/s1, Figure S1: Close up view of interactions between NF-κB p50 subunit loop L1 amino acid side chains (labeled) and DNA bases from NGAL κB DNA packed end-to-end in the p50:(NGAL)_2_ complex crystal structure. Table S1: Results of analysis of structural changes to κB DNA as a consequence of p50 homodimer binding. Figure S2: Results of analysis of structural changes to κB DNA as a consequence of p50 homodimer binding.

## References

[1] Hoffmann A., Levchenko A., Scott M.L., Baltimore D. (2002). The IκB-NF-κB signaling module: Temporal control and selective gene activation. Science.

[2] Xiao C., Ghosh S. (2005). NF-κB, an evolutionarily conserved mediator of immune and inflammatory responses. Adv. Exp. Med. Biol..

[3] Williams L.M., Gilmore T.D. (2020). Looking Down on NF-κB. Mol. Cell. Biol..

[4] Liu T., Zhang L., Joo D., Sun S.C. (2017). NF-κB signaling in inflammation. Signal Transduct. Target. Ther..

[5] Sun S.C. (2017). The non-canonical NF-κB pathway in immunity and inflammation. Nat. Rev. Immunol..

[6] Kaltschmidt C., Greiner J.F.W., Kaltschmidt B. (2021). The transcription factor NF-κB in stem cells and development. Cells.

[7] Taniguchi K., Karin M. (2018). NF-κB, inflammation, immunity and cancer: Coming of age. Nat. Rev. Immunol..

[8] Hayden M.S., Ghosh S. (2008). Shared principles in NF-κB signaling. Cell.

[9] Liou H.C., Nolan G.P., Ghosh S., Fujita T., Baltimore D. (1992). The NF-κB p50 precursor, p105, contains an internal IκB-like inhibitor that preferentially inhibits p50. EMBO J..

[10] Lin L., Ghosh S. (1996). A glycine-rich region in NF-κB p105 functions as a processing signal for the generation of the p50 subunit. Mol. Cell. Biol..

[11] Heissmeyer V., Krappmann D., Wulczyn F.G., Scheidereit C. (1999). NF-κB p105 is a target of IκB kinases and controls signal induction of Bcl-3-p50 complexes. EMBO J..

[12] Cartwright T., Perkins N.D., Caroline L.W. (2016). NFKB1: A suppressor of inflammation, ageing and cancer. FEBS J..

[13] Bates P.W., Miyamoto S. (2004). Expanded nuclear roles for IκBs. Sci STKE.

[14] Trinh D.V., Zhu N., Farhang G., Kim B.J., Huxford T. (2008). The nuclear IκB protein IκBζ specifically binds NF-κB p50 homodimers and forms a ternary complex on κB DNA. J. Mol. Biol..

[15] Yamamoto M., Yamazaki S., Uematsu S., Sato S., Hemmi H., Hoshino K., Kaisho T., Kuwata H., Takeuchi O., Takeshige K. (2004). Regulation of Toll/IL-1-receptor-mediated gene expression by the inducible nuclear protein IκBζ. Nature.

[16] Matsuo S., Yamazaki S., Takeshige K., Muta T. (2007). Crucial roles of binding sites for NF-κB and C/EBPs in IκBζ-mediated transcriptional activation. Biochem. J..

[17] Tartey S., Matsushita K., Vandenbon A., Ori D., Imamura T., Mino T., Standley D.M., Hoffmann J.A., Reichhart J.M., Akira S. (2014). Akirin2 is critical for inducing inflammatory genes by bridging IκBζ and the SWI/SNF complex. EMBO J..

[18] Otwinowski Z., Minor W. (1997). Processing of X-ray diffraction data collected in oscillation mode. Methods Enzymol..

[19] Kabsch W. (2010). XDS. Acta Crystallogr. D Biol. Crystallogr..

[20] Ghosh G., van Duyne G., Ghosh S., Sigler P.B. (1995). Structure of NF-κB p50 homodimer bound to a κB site. Nature.

[21] McCoy A.J., Grosse-Kunstleve R.W., Adams P.D., Winn M.D., Storoni L.C., Read R.J. (2007). Phaser crystallographic software. J. Appl. Crystallogr..

[22] Emsley P., Cowtan K. (2004). Coot: Model-building tools for molecular graphics. Acta Crystallogr. D Biol. Crystallogr..

[23] Adams P.D., Afonine P.V., Bunkoczi G., Chen V.B., Davis I.W., Echols N., Headd J.J., Hung L.W., Kapral G.J., Grosse-Kunstleve R.W. (2010). PHENIX: A comprehensive Python-based system for macromolecular structure solution. Acta Crystallogr. D Biol. Crystallogr..

[24] Williams C.J., Headd J.J., Moriarty N.W., Prisant M.G., Videau L.L., Deis L.N., Verma V., Keedy D.A., Hintze B.J., Chen V.B. (2018). MolProbity: More and better reference data for improved all-atom structure validation. Protein Sci..

[25] Schrödinger L., DeLano W. PyMOL. http://www.pymol.org/pymol.

[26] Li S., Olson W.K., Lu X.J. (2019). Web 3DNA 2.0 for the analysis, visualization, and modeling of 3D nucleic acid structures. Nucleic Acids Res..

[27] Libermann T.A., Baltimore D. (1990). Activation of interleukin-6 gene expression through the NF-κB transcription factor. Mol. Cell. Biol..

[28] Matsusaka T., Fujikawa K., Nishio Y., Mukaida N., Matsushima K., Kishimoto T., Akira S. (1993). Transcription factors NF-IL6 and NF-κB synergistically activate transcription of the inflammatory cytokines, interleukin-6 and interleukin-8. Proc. Natl. Acad. Sci. USA.

[29] Kunsch C., Ruben S.M., Rosen C.A. (1992). Selection of optimal κB/Rel DNA-binding motifs: Interaction of both subunits of NF-κB with DNA is required for transcriptional activation. Mol. Cell. Biol..

[30] Siggers T., Chang A.B., Teixeira A., Wong D., Williams K.J., Ahmed B., Ragoussis J., Udalova I.A., Smale S.T., Bulyk M.L. (2011). Principles of dimer-specific gene regulation revealed by a comprehensive characterization of NF-κB family DNA binding. Nat. Immunol..

[31] Mulero M.C., Wang V.Y., Huxford T., Ghosh G. (2019). Genome reading by the NF-κB transcription factors. Nucleic Acids Res..

[32] Huxford T., Huang D.B., Malek S., Ghosh G. (1998). The crystal structure of the IκBα/NF-κB complex reveals mechanisms of NF-κB inactivation. Cell.

[33] Jacobs M.D., Harrison S.C. (1998). Structure of an IκBα/NF-κB complex. Cell.

[34] Michel F., Soler-Lopez M., Petosa C., Cramer P., Siebenlist U., Müller C.W. (2001). Crystal structure of the ankyrin repeat domain of Bcl-3: A unique member of the IκB protein family. EMBO J..

[35] Malek S., Huang D.B., Huxford T., Ghosh S., Ghosh G. (2003). X-ray crystal structure of an IκBβ:NF-κB p65 homodimer complex. J. Biol. Chem..

[36] Karlsen J.R., Borregaard N., Cowland J.B. (2010). Induction of neutrophil gelatinase-associated lipocalin expression by co-stimulation with interleukin-17 and tumor necrosis factor-α is controlled by IκBζ but neither by C/EBP-β nor C/EBP-δ. J. Biol. Chem..

[37] Cowland J.B., Sorensen O.E., Sehested M., Borregaard N. (2003). Neutrophil gelatinase-associated lipocalin is up-regulated in human epithelial cells by IL-1β, but not by TNF-α. J. Immunol..

[38] Huang D.B., Vu D., Cassiday L.A., Zimmerman J.M., Maher L.J., Ghosh G. (2003). Crystal structure of NF-κB (p50)_2_ complexed to a high-affinity RNA aptamer. Proc. Natl. Acad. Sci. USA.

[39] Ghosh G., Huang D.B., Huxford T. (2004). Molecular mimicry of the NF-κB DNA target site by a selected RNA aptamer. Curr. Opin. Struct. Biol..

[40] Müller C.W., Rey F.A., Sodeoka M., Verdine G.L., Harrison S.C. (1995). Structure of the NF-κB p50 homodimer bound to DNA. Nature.

[41] Müller C.W., Rey F.A., Harrison S.C. (1996). Comparison of two different DNA-binding modes of the NF-κB p50 homodimer. Nat. Struct. Biol..

[42] Huang D.B., Huxford T., Chen Y.Q., Ghosh G. (1997). The role of DNA in the mechanism of NF-κB dimer formation: Crystal structures of the dimerization domains of the p50 and p65 subunits. Structure.

[43] Müller C.W., Harrison S.C. (1995). The structure of the NF-κB p50:DNA-complex: A starting point for analyzing the Rel family. FEBS Lett..

[44] Chen Y.Q., Ghosh S., Ghosh G. (1998). A novel DNA recognition mode by the NF-κB p65 homodimer. Nat. Struct. Biol..

[45] Biancalana M., Natan E., Lenardo M.J., Fersht A.R. (2021). NF-κB Rel subunit exchange on a physiological timescale. Protein Sci..

[46] Phelps C.B., Sengchanthalangsy L.L., Huxford T., Ghosh G. (2000). Mechanism of IκBα binding to NF-κB dimers. J. Biol. Chem..

[47] Zhao B., Barrera L.A., Ersing I., Willox B., Schmidt S.C., Greenfeld H., Zhou H., Mollo S.B., Shi T.T., Takasaki K. (2014). The NF-κB genomic landscape in lymphoblastoid B cells. Cell Rep..

[48] Cao S., Zhang X., Edwards J.P., Mosser D.M. (2006). NF-κB1 (p50) homodimers differentially regulate pro- and anti-inflammatory cytokines in macrophages. J. Biol. Chem..

[49] Cheng C.S., Feldman K.E., Lee J., Verma S., Huang D.B., Huynh K., Chang M., Ponomarenko J.V., Sun S.C., Benedict C.A. (2011). The specificity of innate immune responses is enforced by repression of interferon response elements by NF-κB p50. Sci. Signal..

[50] Chen Y.Q., Sengchanthalangsy L.L., Hackett A., Ghosh G. (2000). NF-κB p65 (RelA) homodimer uses distinct mechanisms to recognize DNA targets. Structure.

[51] Hou S., Guan H., Ricciardi R.P. (2003). Phosphorylation of serine 337 of NF-κB p50 is critical for DNA binding. J. Biol. Chem..

[52] Mulero M.C., Shahabi S., Ko M.S., Schiffer J.M., Huang D.B., Wang V.Y., Amaro R.E., Huxford T., Ghosh G. (2018). Protein cofactors are essential for high-affinity DNA binding by the nuclear factor κB RelA subunit. Biochemistry.

