# Supplementary material for: X-ray Crystallographic Study of Preferred Spacing by the NF-κB p50 Homodimer on κB DNA
Source: Biomolecules. 2023 Aug 26;13(9):1310. doi: 10.3390/biom13091310 (PMC10527052; doi:10.3390/biom13091310)
Supplement: Supplementary file 1 [file biomolecules-13-01310-s001.zip › biomolecules-2551235-supplementary.pdf]

SUPPLEMENTARY FILES

N. Zhu, *et al.*, "X-ray crystallographic study of preferred spacing by the NF-κB p50 homodimer on κB DNA."

Figure S1. Close up view of interactions between NF-κB p50 subunit loop L1 amino acid side chains (labeled) and DNA bases from NGAL κB DNA packed end-to-end in the p50:(NGAL)$_2$ complex crystal structure. A) The interface between two double-stranded 10-mer NGAL κB DNA molecules in asymmetric unit of the p50:(NGAL)$_2$ complex X-ray co-crystal structure is depicted as a ribbon diagram with key amino acid side chains and DNA bases shown as sticks. One NGAL κB DNA (colored magenta and orange, consistent with Figure 1D) is bound by the NF-κB p50 homodimer (green). The other (two-tone grey) packs against the end of the first and contributes only a 5'-G base, which is bound by p50 loop L1 residue H64. B) A similar interaction is observed at the other end where the p50-bound NGAL κB DNA stacks end-to-end with an unbound NGAL κB DNA from a neighboring asymmetric unit, which contributes a 5'-G to the complex. The consequence is that one NF-κB p50 homodimer binds to one blunt-ended 10-mer NGAL κB DNA with 12-bp spacing.

Table S1. Results of analysis of structural changes to κB DNA as a consequence of p50 homodimer binding. Ideal B-DNA models for each of the κB DNA sequences employed in the X-ray crystallographic studies were generated in WinCoot [22]. Base pair geometric parameters (shear, stretch, stagger, buckle, propeller, and opening) are measured for the 17-mer IL-6 κB DNA in its p50-bound and ideal B-DNA conformations as well as their differences (Page 1). An analysis of major and minor groove width at each base pair for p50-bound and ideal B-DNA conformations of the 17-mer IL-6 κB DNA (Page 2). Similar analysis was performed on both p50-bound ("Central DNA"), p50-unbound ("Terminal DNA"), and Ideal B-DNA conformations of blunt-ended 10-mer NGAL κB DNA (Pages 3 and 4) and with p50-bound and ideal B-DNA conformations of 16-mer Test κB-like DNA (Pages 5 and 6). Analysis was performed with Web 3DNA 2.0 at https://web.x3dna.org [26]. Details on the definitions of each of the parameters measured are provided at the website.

Figure S2. The dimerization domains of p50 homodimer contact DNA differently as a consequence of 11- or 12-bp spacing. A) Ribbon diagram representation of the p50:IL-6 κB DNA complex X-ray co-crystal structure. The two p50 subunits are green and the double-stranded DNA is pink and yellow (consistent with Figure 1C). A similar representation of the p50:NGAL κB DNA complex, with p50 in blue and DNA strands as magenta and orange, is superimposed via least-squares fit of the dimerization domains. The pseudo-dyad axis of symmetry is shown as a black line. B) A close up view of the 2-fold symmetry axis as it passes through the DNA reveals that is passes between A_0:T_0 of IL-6 κB DNA and between $A_{-0}T_{+0}$:$T_{-0}A_{+0}$ of NGAL κB DNA. Therefore, despite the dimerization domains being in the same place, the DNA they contact is in a different position. C) Close up view of superimposed p50 dimerization domains from Figure 5B, with DNA ribose-phosphate backbone-contacting amino acid residues from the dimerization domains depicted as sticks. The amino acids, like the dimerization domains, overlay with one another but interact with the bound IL-6 and NGAL κB DNA molecules via distinct modes.

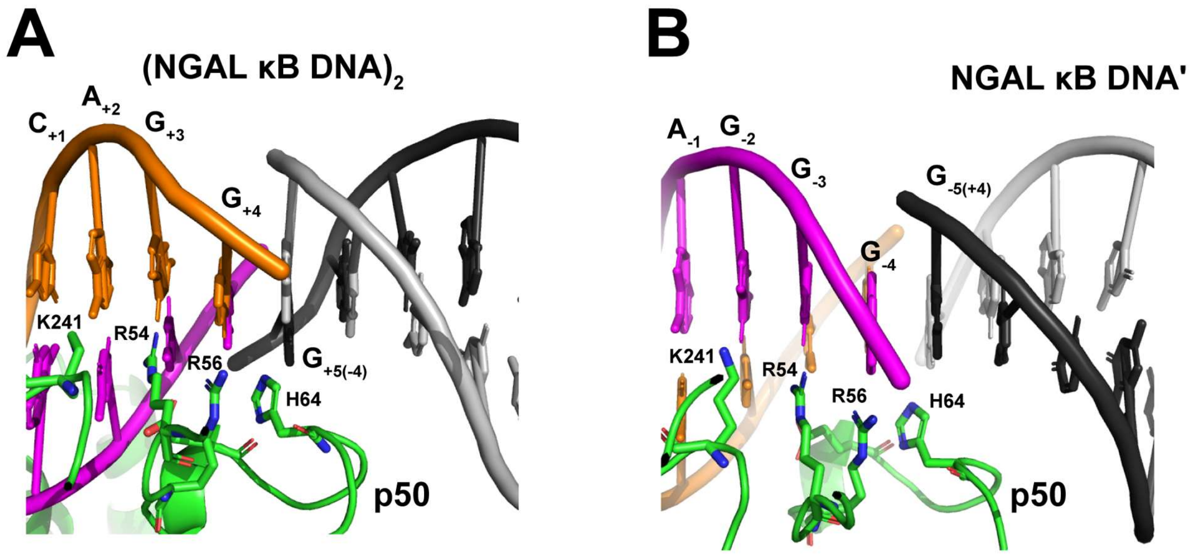
A
(NGAL κB DNA)$_2$
C$_{+1}$
A$_{+2}$
G$_{+3}$
G$_{+4}$
K241
R54
R56
G$_{+5(-4)}$
H64
p50
B
NGAL κB DNA'
A$_{-1}$
G$_{-2}$
G$_{-3}$
G$_{-5(+4)}$
G$_{-4}$
K241
R54
R56
H64
p50

Figure S1

IL-6 κB DNA bound by p50							
Pair ID	Base Pair	Shear	Stretch	Stagger	Buckle	Propeller	Opening
1	T-A	-0.15	-0.14	-0.38	-0.56	-3.01	0.13
2	G-C	-0.24	-0.12	-0.08	-3.45	-6.42	4.02
3	T-A	-0.05	-0.11	0.13	-0.03	-6.78	1.34
4	G-C	-0.16	-0.17	0.33	0.06	-4.48	-2.22
5	G-C	-0.22	-0.18	-0.02	2.08	-1.88	0.98
6	G-C	-0.2	-0.16	0.2	5.39	-6.49	0.39
7	A-T	0.06	-0.13	0.27	4.2	-11.16	1.73
8	T-A	-0.3	-0.2	0.43	-1.49	-16.97	-0.23
9	T-A	-0.19	-0.18	0.22	-2.44	-18.53	3.29
10	T-A	-0.15	-0.15	-0.02	-3.46	-16.04	1.86
11	T-A	-0.14	-0.18	-0.29	-7.82	-11.49	3.21
12	C-G	0.21	-0.13	-0.12	-3.93	-5.34	-0.25
13	C-G	0.23	-0.19	-0.32	2.6	-1.55	2.28
14	C-G	0.2	-0.17	0.18	-1.63	-4.03	-0.38
15	A-T	0.05	-0.14	0.05	-3.84	-2.85	2.99
16	T-A	-0.07	-0.17	0.3	3.89	-1.65	2.73
17	G-C	-0.16	-0.15	-0.27	6.56	0.31	-2.47

Ideal B-DNA versus IL-6 κB DNA							
Pair ID	Base Pair	Shear	Stretch	Stagger	Buckle	Propeller	Opening
1	T-A	0.1	-0.17	0.08	-0.09	-14.46	-1.1
2	G-C	-0.3	-0.2	0.11	0.31	-14.67	0.24
3	T-A	0.1	-0.17	0.08	-0.08	-14.46	-1.09
4	G-C	-0.3	-0.2	0.11	0.31	-14.67	0.22
5	G-C	-0.3	-0.2	0.11	0.31	-14.67	0.24
6	G-C	-0.3	-0.2	0.11	0.31	-14.67	0.24
7	A-T	-0.1	-0.17	0.08	0.08	-14.46	-1.09
8	T-A	0.1	-0.17	0.08	-0.08	-14.46	-1.1
9	T-A	0.1	-0.17	0.08	-0.08	-14.46	-1.09
10	T-A	0.1	-0.17	0.08	-0.08	-14.46	-1.1
11	T-A	0.1	-0.17	0.08	-0.08	-14.46	-1.1
12	C-G	0.3	-0.2	0.11	-0.31	-14.68	0.22
13	C-G	0.3	-0.2	0.11	-0.31	-14.68	0.23
14	C-G	0.3	-0.2	0.11	-0.31	-14.69	0.23
15	A-T	-0.1	-0.17	0.08	0.09	-14.46	-1.09
16	T-A	0.1	-0.17	0.08	-0.09	-14.46	-1.09
17	G-C	-0.3	-0.2	0.11	0.32	-14.69	0.25

Difference in Base Geometries							
Pair ID	Base Pair	Shear	Stretch	Stagger	Buckle	Propeller	Opening
1	T-A	0.25	0.03	0.46	0.47	11.45	1.23
2	G-C	0.06	0.08	0.19	3.76	8.25	3.78
3	T-A	0.15	0.06	0.05	0.05	7.68	2.43
4	G-C	0.14	0.03	0.22	0.25	10.19	2.44
5	G-C	0.08	0.02	0.13	1.77	12.79	0.74
6	G-C	0.1	0.04	0.09	5.08	8.18	0.15
7	A-T	0.16	0.04	0.19	4.12	3.3	2.82
8	T-A	0.4	0.03	0.35	1.41	2.51	0.87
9	T-A	0.29	0.01	0.14	2.36	4.07	4.38
10	T-A	0.25	0.02	0.1	3.38	1.58	2.96
11	T-A	0.24	0.01	0.37	7.74	2.97	4.31
12	C-G	0.09	0.07	0.23	3.62	9.34	0.47
13	C-G	0.07	0.01	0.43	2.91	13.13	2.05
14	C-G	0.1	0.03	0.07	1.32	10.66	0.61
15	A-T	0.15	0.03	0.03	3.93	11.61	4.08
16	T-A	0.17	0	0.22	3.98	12.81	3.82
17	G-C	0.14	0.05	0.38	6.24	15	2.72

IL-6 κB DNA bound by p50					
		Minor Groove		Major Groove	
Pair ID	Base Pair	P-P	Refined	P-P	Refined
1	TG/CA	---	---	---	---
2	GT/AC	---	---	---	---
3	TG/CA	13.7	---	18.3	---
4	GG/CC	12.7	12.7	20.7	20.7
5	GG/CC	11.8	11.8	22.3	22.2
6	GA/TC	10.6	10.6	20.8	20.7
7	AT/AT	9.5	9.5	18.3	18
8	TT/AA	9.9	9.8	16.6	16.2
9	TT/AA	9.9	9.9	16.8	16.4
10	TT/AA	9.8	9.7	18.1	18
11	TC/GA	11	11	21.1	21
12	CC/GG	11.9	11.9	22.4	22.2
13	CC/GG	12.5	12.4	21.4	21.3
14	CA/TG	14.1	---	18.4	---
15	AT/AT	---	---	---	---
16	TG/CA	---	---	---	---

Ideal B-DNA versus IL-6 κB DNA					
		Minor Groove		Major Groove	
Pair ID	Base Pair	P-P	Refined	P-P	Refined
1	TG/CA	---	---	---	---
2	GT/AC	---	---	---	---
3	TG/CA	11.7	---	17.2	---
4	GG/CC	11.7	11.7	17.2	17.2
5	GG/CC	11.7	11.7	17.2	17.2
6	GA/TC	11.7	11.7	17.2	17.2
7	AT/AT	11.7	11.7	17.2	17.2
8	TT/AA	11.7	11.7	17.2	17.2
9	TT/AA	11.7	11.7	17.2	17.2
10	TT/AA	11.7	11.7	17.2	17.2
11	TC/GA	11.7	11.7	17.2	17.2
12	CC/GG	11.7	11.7	17.2	17.2
13	CC/GG	11.7	11.7	17.2	17.2
14	CA/TG	11.7	---	17.2	---
15	AT/AT	---	---	---	---
16	TG/CA	---	---	---	---

Differences in Minor and Major Grooves					
		Minor Groove		Major Groove	
Pair ID	Base Pair	P-P	Refined	P-P	Refined
1	TG/CA	---	---	---	---
2	GT/AC	---	---	---	---
3	TG/CA	2	---	1.1	---
4	GG/CC	1	1	3.5	3.5
5	GG/CC	0.1	0.1	5.1	5
6	GA/TC	1.1	1.1	3.6	3.5
7	AT/AT	2.2	2.2	1.1	0.8
8	TT/AA	1.8	1.9	0.6	1
9	TT/AA	1.8	1.8	0.4	0.8
10	TT/AA	1.9	2	0.9	0.8
11	TC/GA	0.7	0.7	3.9	3.8
12	CC/GG	0.2	0.2	5.2	5
13	CC/GG	0.8	0.7	4.2	4.1
14	CA/TG	2.4	---	1.2	---
15	AT/AT	---	---	---	---
16	TG/CA	---	---	---	---

Table S1-page 2

Ideal B-DNA versus NGAL κB-DNA							
Pair ID	Base Pair	Shear	Stretch	Stagger	Buckle	Propeller	Opening
1	G-C	-0.3	-0.2	0.11	0.33	-14.67	0.23
2	G-C	-0.3	-0.2	0.11	0.33	-14.67	0.22
3	G-C	-0.3	-0.2	0.11	0.33	-14.67	0.23
4	A-T	-0.1	-0.17	0.08	0.08	-14.47	-1.11
5	A-T	-0.1	-0.17	0.08	0.08	-14.47	-1.11
6	T-A	0.1	-0.17	0.08	-0.11	-14.48	-1.1
7	G-C	-0.3	-0.2	0.11	0.33	-14.67	0.23
8	T-A	0.1	-0.17	0.08	-0.11	-14.48	-1.1
9	C-G	0.3	-0.2	0.11	-0.33	-14.67	0.23
10	C-G	0.3	-0.2	0.11	-0.33	-14.67	0.23

NGAL κB DNA bound by p50							
Pair ID	Base Pair	Shear	Stretch	Stagger	Buckle	Propeller	Opening
Central DNA							
1	G-C	-0.2	-0.14	0.16	2.27	-2.07	0.05
2	G-C	-0.2	-0.2	0.43	3.63	-5.91	-1.57
3	G-C	-0.17	-0.17	0.19	2.64	-8.48	0.65
4	A-T	0.09	-0.2	0.17	4.34	-7.17	-1.47
5	A-T	0.13	-0.17	0.29	1.04	-16.02	-1.52
6	T-A	-0.22	-0.18	0.41	-6.15	-17.05	-4.01
7	G-C	-0.12	-0.13	0.21	-8.09	-3.79	0.05
8	T-A	-0.23	-0.14	0.08	-5.07	-5.19	0.09
9	C-G	0.17	-0.19	0.11	-1.82	-6.53	-0.21
10	C-G	0.2	-0.19	-0.08	-0.22	-4.19	1.6
Terminal DNA							
1	G-C	-0.19	-0.14	0.13	0.75	-2.31	-0.83
2	G-C	-0.21	-0.15	0.21	-0.67	-2.45	-1.3
3	G-C	-0.2	-0.13	0.32	5.31	-1.81	-0.03
4	A-T	0.14	-0.17	0.25	3.47	-6.19	-0.24
5	A-T	0.1	-0.17	0.01	-1.68	-8.94	4.93
6	T-A	-0.14	-0.05	-0.04	-3.09	-7.06	-6.24
7	G-C	-0.2	-0.16	-0.18	-2.28	-0.25	0.36
8	T-A	-0.06	-0.18	0.1	-2.45	-2.53	1.4
9	C-G	0.16	-0.18	-0.17	3	2.41	-0.62
10	C-G	0.23	-0.13	-0.04	-0.12	0.92	1.88

Difference in Base Geometries							
Pair ID	Base Pair	Shear	Stretch	Stagger	Buckle	Propeller	Opening
Central DNA							
1	G-C	0.1	0.06	0.05	1.94	12.6	0.18
2	G-C	0.1	0	0.32	3.3	8.76	1.79
3	G-C	0.13	0.03	0.08	2.31	6.19	0.42
4	A-T	0.19	0.03	0.09	4.26	7.3	0.36
5	A-T	0.23	0	0.21	0.96	1.55	0.41
6	T-A	0.32	0.01	0.33	6.04	2.57	2.91
7	G-C	0.18	0.07	0.1	8.42	10.88	0.18
8	T-A	0.33	0.03	0	4.96	9.29	1.19
9	C-G	0.13	0.01	0	1.49	8.14	0.44
10	C-G	0.1	0.01	0.19	0.11	10.48	1.37
Terminal DNA							
1	G-C	0.11	0.06	0.02	0.42	12.36	1.06
2	G-C	0.09	0.05	0.1	1	12.22	1.52
3	G-C	0.1	0.07	0.21	4.98	12.86	0.26
4	A-T	0.24	0	0.17	3.39	8.28	0.87
5	A-T	0.2	0	0.07	1.76	5.53	6.04
6	T-A	0.24	0.12	0.12	2.98	7.42	5.14
7	G-C	0.1	0.04	0.29	2.61	14.42	0.13
8	T-A	0.16	0.01	0.02	2.34	11.95	2.5
9	C-G	0.14	0.02	0.28	3.33	17.08	0.85
10	C-G	0.07	0.07	0.15	0.21	15.59	1.65

NGAL κB DNA bound by p50					
		Minor Groove		Major Groove	
Central DNA					
Pair ID	Base Pair	P-P	Refined	P-P	Refined
1	GG/CC	---	---	---	---
2	GG/CC	---	---	---	---
3	GA/TC	10.8	---	19.3	---
4	AA/TT	9.7	9.7	17.9	17.9
5	AT/AT	9.5	9.5	16.1	16
6	TG/CA	9.3	9.3	18.3	18.3
7	GT/AC	10.4	---	20.1	---
8	TC/GA	---	---	---	---
9	CC/GG	---	---	---	---
Terminal DNA					
1	GG/CC	---	---	---	---
2	GG/CC	---	---	---	---
3	GA/TC	12.9	---	19.4	---
4	AA/TT	12.7	12.6	19.9	19.3
5	AT/AT	12.7	12.5	20.2	19.5
6	TG/CA	12.4	12	19.6	18.2
7	GT/AC	12.9	---	17.9	---
8	TC/GA	---	---	---	---
9	CC/GG	---	---	---	---

Ideal B-DNA versus NGAL κB-DNA					
		Minor Groove		Major Groove	
Pair ID	Base Pair	P-P	Refined	P-P	Refined
1	GG/CC	---	---	---	---
2	GG/CC	---	---	---	---
3	GA/TC	11.7	---	17.2	---
4	AA/TT	11.7	11.7	17.2	17.2
5	AT/AT	11.7	11.7	17.2	17.2
6	TG/CA	11.7	11.7	17.2	17.2
7	GT/AC	11.7	---	17.2	---
8	TC/GA	---	---	---	---
9	CC/GG	---	---	---	---

Differences in Minor and Major Grooves					
		Minor Groove		Major Groove	
Central DNA					
Pair ID	Base Pair	P-P	Refined	P-P	Refined
1	GG/CC				
2	GG/CC				
3	GA/TC	0.9		2.1	
4	AA/TT	2	2	0.7	0.7
5	AT/AT	2.2	2.2	1.1	1.2
6	TG/CA	2.4	2.4	1.1	1.1
7	GT/AC	1.3		2.9	
8	TC/GA				
9	CC/GG				
Terminal DNA					
1	GG/CC				
2	GG/CC				
3	GA/TC	1.2		2.2	
4	AA/TT	1	0.9	2.7	2.1
5	AT/AT	1	0.8	3	2.3
6	TG/CA	0.7	0.3	2.4	1
7	GT/AC	1.2		0.7	
8	TC/GA				
9	CC/GG				

Test κB DNA bound by p50							
Pair ID	Base Pair	Shear	Stretch	Stagger	Buckle	Propeller	Opening
1	C-G	0.22	-0.14	0.11	-2.35	-5.33	2.64
2	A-T	0.09	-0.15	-0.03	-2.08	-3.21	1.88
3	G-C	-0.16	-0.14	0.18	3.97	-8.16	-1.74
4	G-C	-0.23	-0.25	-0.96	-5.99	-2.11	1.69
5	G-C	-0.21	-0.19	-0.54	0.12	-0.43	0.59
6	G-C	-0.2	-0.14	-0.19	4.85	-5.35	2.83
7	A-T	0.08	-0.14	-0.31	-2.42	-7.38	3.87
8	A-T	0.07	-0.15	-0.45	-4.34	-11.89	4
9	T-A	-0.05	-0.17	-0.52	-2.71	-10.95	6.57
10	T-A	-0.18	-0.22	-0.67	-4.08	-9.72	1.17
11	C-G	0.22	-0.2	-0.61	-3.84	-6.09	1.8
12	C-G	0.22	-0.17	-0.36	-2.54	-6.06	1.8
13	C-G	0.2	-0.13	-0.33	-4.04	-5.42	0.58
14	C-G	0.21	-0.18	-0.63	2.85	-7.18	3.15
15	T-A	-0.08	-0.1	-0.46	6.45	-13.28	1.09
16	C-G	-1.63	0.2	0.55	1.31	-23.59	9.86

Ideal B-DNA versus Test κB DNA							
Pair ID	Base Pair	Shear	Stretch	Stagger	Buckle	Propeller	Opening
1	T-A	0.1	-0.17	0.08	-0.08	-14.47	-1.1
2	C-G	0.3	-0.2	0.11	-0.33	-14.67	0.23
3	A-T	-0.1	-0.17	0.08	0.11	-14.47	-1.11
4	G-C	-0.3	-0.2	0.11	0.33	-14.67	0.22
5	G-C	-0.3	-0.2	0.11	0.33	-14.67	0.24
6	G-C	-0.3	-0.2	0.11	0.33	-14.67	0.22
7	G-C	-0.3	-0.2	0.11	0.33	-14.67	0.23
8	A-T	-0.1	-0.17	0.08	0.11	-14.47	-1.11
9	A-T	-0.1	-0.17	0.08	0.11	-14.47	-1.1
10	T-A	0.1	-0.17	0.08	-0.08	-14.47	-1.1
11	T-A	0.1	-0.17	0.08	-0.08	-14.47	-1.1
12	C-G	0.3	-0.2	0.11	-0.33	-14.67	0.23
13	C-G	0.3	-0.2	0.11	-0.33	-14.67	0.24
14	C-G	0.3	-0.2	0.11	-0.33	-14.67	0.23
15	C-G	0.3	-0.2	0.11	-0.33	-14.67	0.24
16	T-A	0.1	-0.17	0.08	-0.08	-14.47	-1.09
17	C-G	0.3	-0.2	0.11	-0.33	-14.67	0.23

Difference in Base Geometries							
Pair ID	Base Pair	Shear	Stretch	Stagger	Buckle	Propeller	Opening
1	T-A	0.12	0.03	0.03	2.27	9.14	3.74
2	C-G	0.21	0.05	0.14	1.75	11.46	1.65
3	A-T	0.06	0.03	0.1	3.86	6.31	0.63
4	G-C	0.07	0.05	1.07	6.32	12.56	1.47
5	G-C	0.09	0.01	0.65	0.21	14.24	0.35
6	G-C	0.1	0.06	0.3	4.52	9.32	2.61
7	G-C	0.38	0.06	0.42	2.75	7.29	3.64
8	A-T	0.17	0.02	0.53	4.45	2.58	5.11
9	A-T	0.05	0	0.6	2.82	3.52	7.67
10	T-A	0.28	0.05	0.75	4	4.75	2.27
11	T-A	0.12	0.03	0.69	3.76	8.38	2.9
12	C-G	0.08	0.03	0.47	2.21	8.61	1.57
13	C-G	0.1	0.07	0.44	3.71	9.25	0.34
14	C-G	0.09	0.02	0.74	3.18	7.49	2.92
15	C-G	0.38	0.1	0.57	6.78	1.39	0.85
16	T-A	1.73	0.37	0.47	1.39	9.12	10.95
17	C-G	0.3	0.2	0.11	0.33	14.67	0.23

Table S1-page 5

Test κB DNA bound by p50					
		Minor Groove		Major Groove	
Pair ID	Base Pair	P-P	Refined	P-P	Refined
1	CA/TG	---	---	---	---
2	AG/CT	---	---	---	---
3	GG/CC	12.5	---	21.5	---
4	GG/CC	12.4	12.4	22.2	22.1
5	GG/CC	11.8	11.8	20.3	20.3
6	GA/TC	10.1	10	18.3	18
7	AA/TT	10	9.9	16.7	16.3
8	AT/AT	10.2	10.1	16.1	15.7
9	TT/AA	10	10	17.5	17.1
10	TC/GA	10.8	10.8	20	20
11	CC/GG	11.1	11.1	21.4	21.4
12	CC/GG	11.6	11.6	20.3	20.1
13	CC/GG	12.4	---	17.1	---
14	CT/AG	---	---	---	---
15	TC/GA	---	---	---	---

Ideal B-DNA versus Test κB DNA					
		Minor Groove		Major Groove	
Pair ID	Base Pair	P-P	Refined	P-P	Refined
1	TC/GA	---	---	---	---
2	CA/TG	---	---	---	---
3	AG/CT	11.7	---	17.2	---
4	GG/CC	11.7	11.7	17.2	17.2
5	GG/CC	11.7	11.7	17.2	17.2
6	GG/CC	11.7	11.7	17.2	17.2
7	GA/TC	11.7	11.7	17.2	17.2
8	AA/TT	11.7	11.7	17.2	17.2
9	AT/AT	11.7	11.7	17.2	17.2
10	TT/AA	11.7	11.7	17.2	17.2
11	TC/GA	11.7	11.7	17.2	17.2
12	CC/GG	11.7	11.7	17.2	17.2
13	CC/GG	11.7	11.7	17.2	17.2
14	CC/GG	11.7	---	17.2	---
15	CT/AG	---	---	---	---
16	TC/GA	---	---	---	---

Differences in Minor and Major Grooves					
		Minor Groove		Major Groove	
Pair ID	Base Pair	P-P	Refined	P-P	Refined
1	CA/TG	---	---	---	---
2	AG/CT	---	---	---	---
3	GG/CC	0.8	---	4.3	---
4	GG/CC	0.7	0.7	5	4.9
5	GG/CC	0.1	0.1	3.1	3.1
6	GA/TC	1.6	1.7	1.1	0.8
7	AA/TT	1.7	1.8	0.5	0.9
8	AT/AT	1.5	1.6	1.1	1.5
9	TT/AA	1.7	1.7	0.3	0.1
10	TC/GA	0.9	0.9	2.8	2.8
11	CC/GG	0.6	0.6	4.2	4.2
12	CC/GG	0.1	0.1	3.1	2.9
13	CC/GG	0.7	---	0.1	---
14	CT/AG	---	---	---	---
15	TC/GA	---	---	---	---
16	TC/GA	---	---	---	---

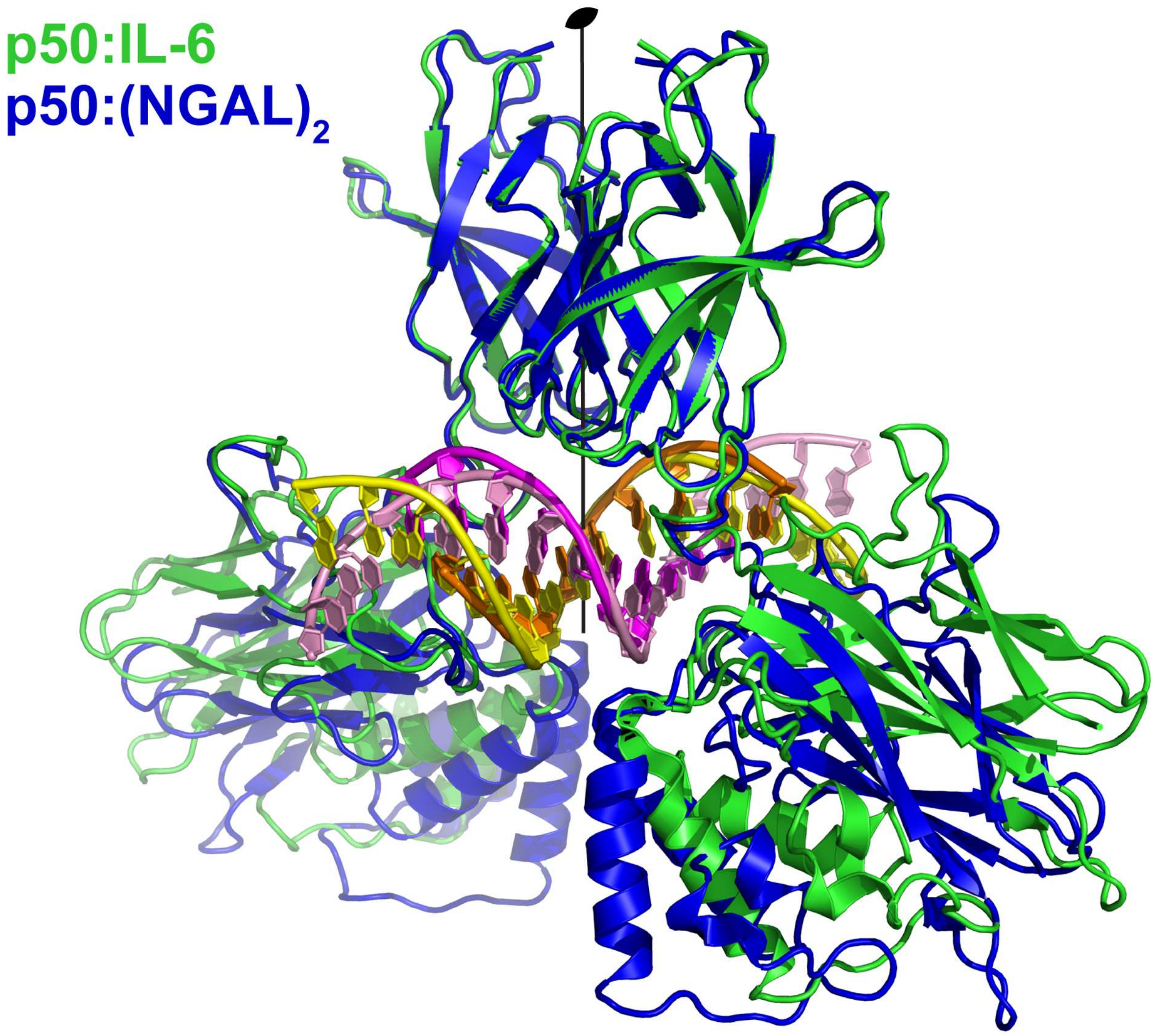

Figure S2A

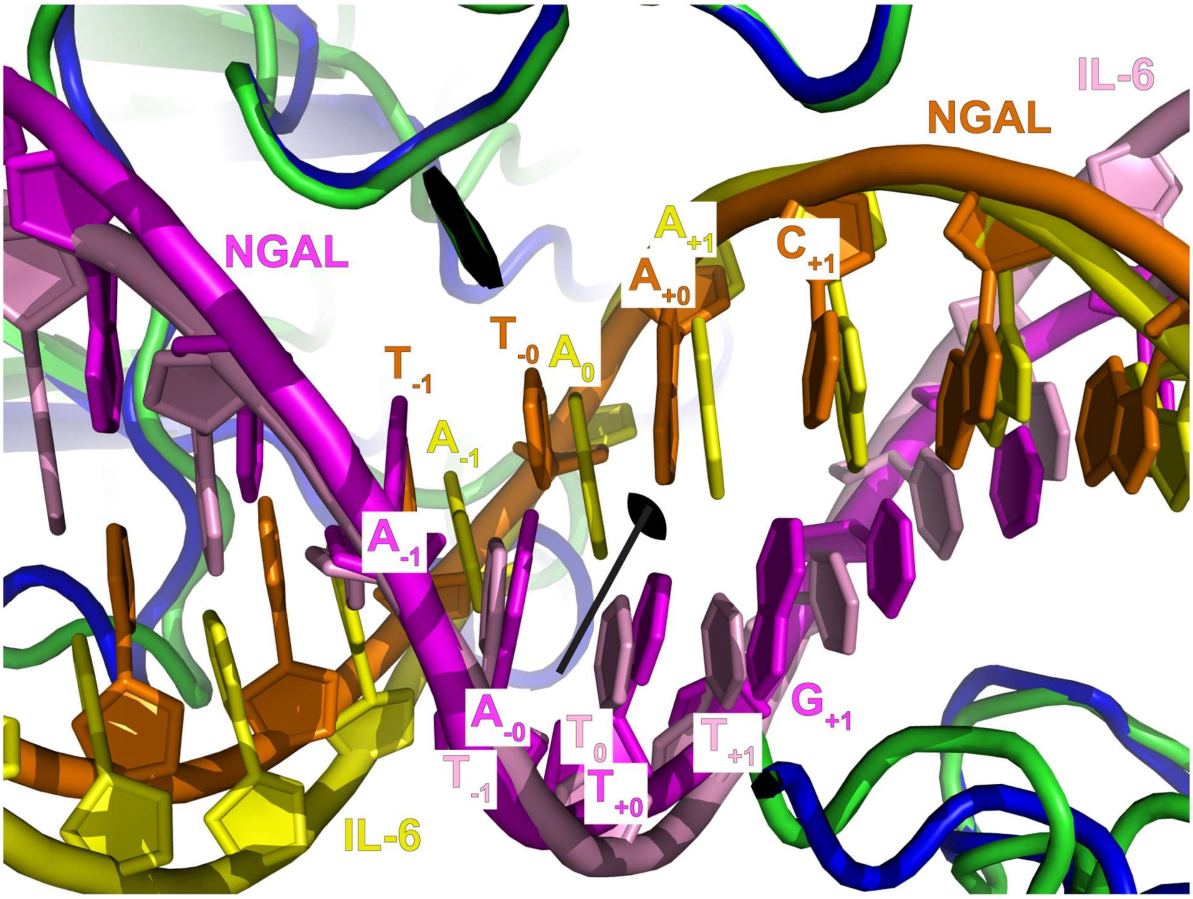

Figure S2B

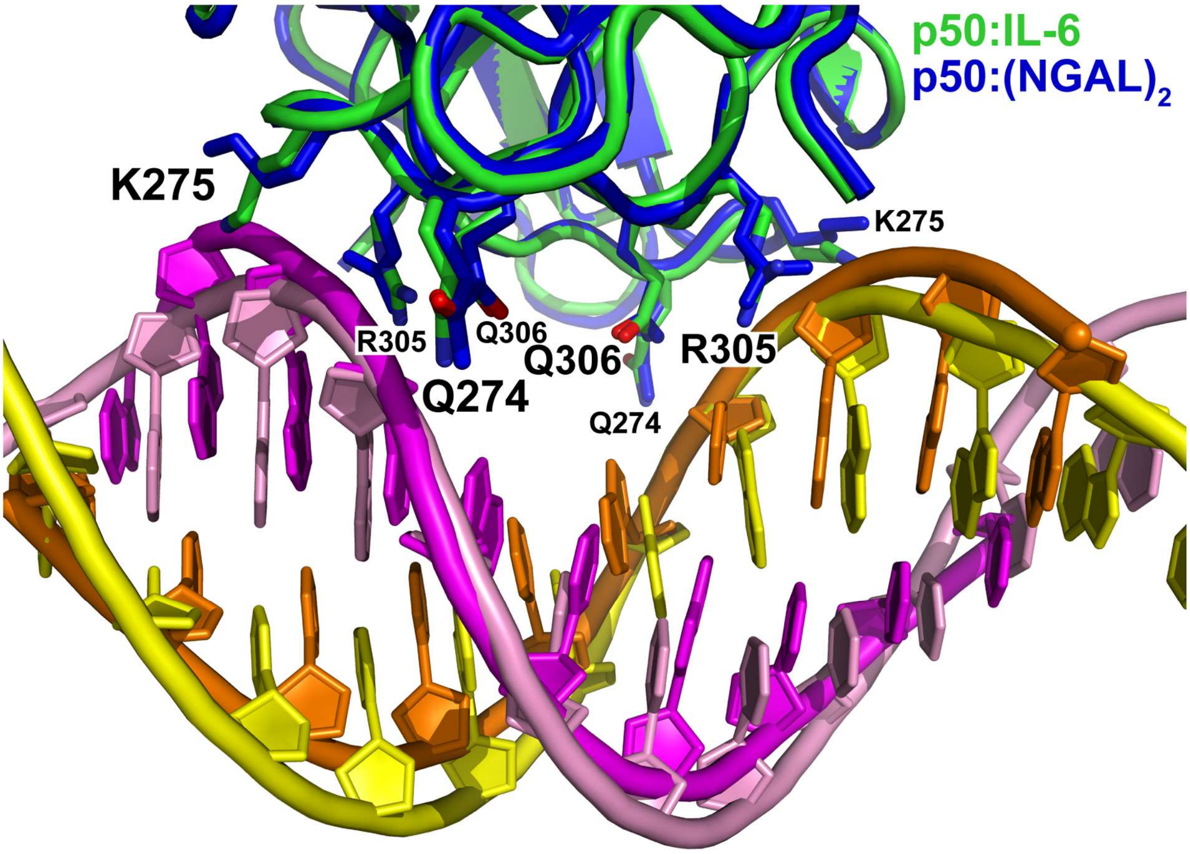

Figure S2C
